# Genomic Analysis of Early Monkeypox Virus Outbreak Strains, Washington, USA

**DOI:** 10.3201/eid2903.221446

**Published:** 2023-03

**Authors:** Pavitra Roychoudhury, Jaydee Sereewit, Hong Xie, Ethan Nunley, Shah M. Bakhash, Nicole A.P. Lieberman, Alexander L. Greninger

**Affiliations:** University of Washington, Seattle, Washington, USA (P. Roychoudhury, J. Sereewit, H. Xie, E. Nunley, S.M. Bakhash, N.A.P. Lieberman, A.L. Greninger);; Fred Hutchinson Cancer Research Center, Seattle (P. Roychoudhury, A.L. Greninger)

**Keywords:** monkeypox virus, viruses, mpox, sexually transmitted infections, zoonoses, sequencing, genomics, Washington, United States

## Abstract

We conducted a genomic analysis of monkeypox virus sequences collected early in the 2022 outbreak, during July–August , in Washington, USA. Using 109 viral genomes, we found low overall genetic diversity, multiple introductions into the state, ongoing community transmission, and potential for co-infections by multiple strains.

The World Health Organization declared the 2022 mpox (formerly monkeypox) outbreak a public health emergency of international concern on July 23, 2022, after cases were identified in nearly 80 countries (*1*). By August 26, 2022, a total of 411 mpox cases had been confirmed in Washington, USA (*2*), and 17,432 cases had been confirmed in the United States (https://www.cdc.gov/poxvirus/monkeypox/response/2022/us-map.html). 

Viral whole-genome sequencing (WGS) can augment contact tracing efforts and identify emerging variants, which potentially could affect infectivity, virulence, vaccine escape, and treatment resistance. By late August 2022, Washington had deposited more monkeypox virus (MPXV) sequences into public databases than any other state in the country. Here, we describe the Washington outbreak by using 109 MPXV genomes collected in the state.

We attempted WGS on 140 residual clinical specimens, primarily lesion swabs, that were PCR-positive for MPXV and had a cycle threshold (Ct) value <31 (range 15.9–30.4). We performed sequencing by using a hybridization probe-capture–based approach, as previously described (*3*), and probes designed by using the MPXV 2022/MA001 strain (Genbank accession no. ON563414) (Appendix). We generated consensus genomes by using Revica (https://github.com/greninger-lab/revica), a custom pipeline that performs trimming, filtering, and iterative remapping (Appendix). Sequences with <1% ambiguous bases (Ns) were deposited to GenBank under BioProject accession no. PRJNA862948 (Appendix Table). We used Augur, Auspice, and Nextclade to perform phylogenetic analysis (*4*,*5*), and we used UShER (*6*) to perform phylogenetic placement on a global tree (Appendix). This study was approved by the University of Washington Institutional Review Board STUDY00000408.

The analysis comprised a total of 109 sequences from 98 persons whose specimens were collected during July 6–August 19, 2022, primarily from King and Pierce Counties. Of the 98 patients, 90 (91.84%) were male and 1 (1.02%) female; 7 (7.14%) had unknown or undeclared sex. Median age at specimen collection was 36.0 (range 19–57) years.

We identified multiple identical genomes from different persons, suggesting ongoing community transmission (Figure, panel A). All 109 genomes fell within the predominant 2022 outbreak lineage B.1 (*7*), and sublineages included B.1.1 (n = 18), B.1.2 (n = 6), B.1.3 (n = 10), B.1.4 (n = 2), and B.1.8 (n = 2), suggesting separate MPXV introductions into the state. Among sublineages, we identified the nearest neighbor sequences from Germany (B.1.1); Connecticut, USA (B.1.2); Canada (B.1.4); Florida, USA (B.1.8); and multiple countries in Europe (B.1.3) (Appendix).

**Figure:**
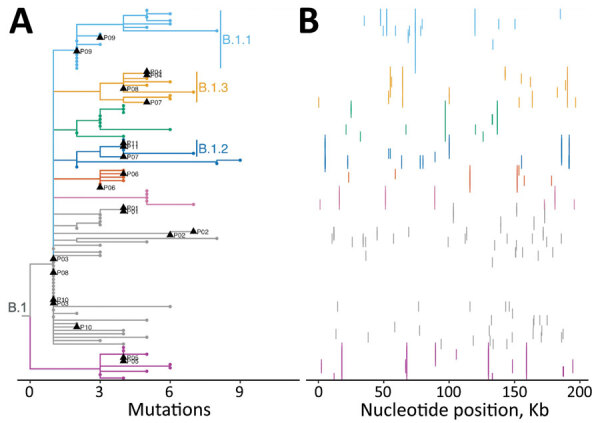
Phylogenomic analysis of 109 early monkeypox virus outbreak strains, Washington, USA. A) Phylogenetic tree showing that all Washington sequences fall within the major outbreak lineage B.1. The many identical sequences suggest community transmission; distinct sublineages suggest multiple MPXV introductions into the state. Black triangles indicate sequences from multiple swabs from the same patient, which were available for 11 persons, patients P01–P11. Clades with >5 sequences were assigned a color for tips and branches, and have text labels for the major sublineages, B.1.1, B.1.2, and B.1.3. All other tips and smaller clades are indicated in gray. B) Single nucleotide polymorphisms from each sample in panel A arrayed across the MPXV genome. Colors correspond to lineage coloring in panel A. MPXV, monkeypox virus.

Overall, we observed low genetic diversity and a median of 1 aa (range 0–7 aa) mutation (substitutions or deletions) across the genome relative to the B.1 ancestor (genome MPXV_USA_2022_MA001; Genbank accession no. ON563414). We identified 138 unique SNPs across the genome in the 109 sequences (Figure, panel B), producing 66 unique mutations (amino acid substitutions or deletions) in 51 genes. Of these, 5 unique aa substitutions (S553N, A1232V, D1546N, D1604N, and S1633L) occurred in surface glycoprotein OPG210, and 3 (E306K, D441Y, and E553K) in OPG189, which encodes one of several ankyrin-repeat proteins (Appendix Figure 1). We noted an abundance of G to A and C to T nucleotide substitutions (Appendix Figure 2), indicative of apolipoprotein B mRNA editing catalytic polypeptide-like3 activity consistent with other reports (*8*). We did not identify any substitutions or deletions in OPG057, a membrane glycoprotein homologous to F13L in vaccinia virus and the putative target of the therapeutic antiviral tecovirimat currently used to treat mpox (*9*).

Sequences from multiple swabs from the same person at the same time point had a median pairwise nucleotide difference of 1 (range 0–10 for 11 sample pairs) outside of labile tandem repeat regions (*10*). We observed even greater similarity in protein sequences with 0 (range 0–6) median pairwise aa differences. Among sample pairs from 3 patients, patient 06 (P06) had 1 aa difference, P07 had 6 aa differences, and P08 had 2 aa differences. Relative to the B.1 ancestral strain MA001, one of the P06 pair featured a V195I mutation in OPG079. One of the P08 pair had synonymous mutations in OPG073 and OPG083, and an OPG003:R84K substitution. Finally, differences in repeat samples from P07 suggest possible co-infection with strains from the B.1.2 and B.1.3 lineages, consistent with the patient’s clinical history indicating multiple sexual partners. Relative to the MA001 B.1 reference strain, one of the P07 samples had synonymous mutations in OPG083 and OPG189, OPG180:D325N, and OPG016:R84K. The other of the P07 pair shared none of those SNPs, but had OPG015:V261A, OPG109:I66V, and the B.1.2-defining OPG210:D1604N. These mutations remained after re-extracting and re-sequencing the original specimens and, compared with interhost variation, suggest the possibility of co-infection with different MPXV strains (Appendix Table).

Overall, our data showed ongoing community MPXV transmission in Washington. The limited MPXV genetic diversity makes it challenging to use WGS data for contact tracing. However, continued genomic surveillance will be crucial for tracking viral evolution and identifying mutations associated with vaccine escape or antiviral treatment resistance.

Appendix Additional information on genomic analysis of early mpox virus outbreak strains, Washington, United States.
