# Supplementary material for: Genomic Analysis of Early Monkeypox Virus Outbreak Strains, Washington, USA
Source: Emerg Infect Dis. 2023 Mar;29(3):644–6. doi: 10.3201/eid2903.221446 (PMC9973680; doi:10.3201/eid2903.221446)
Supplement: Appendix — Additional information on genomic analysis of early mpox virus outbreak strains, Washington, United States. [file 22-1446-Techapp-s1.pdf]

Article DOI: https://doi.org/10.3201/eid2903.221446

Genomic Analysis of Early Monkeypox Virus Outbreak Strains, Washington, United States

Appendix

Additional Methods

Sequencing Approach

DNA was extracted by using the MagNA Pure 96 DNA and Viral NA Small Volume Kit (Roche, https://www.roche.com), and sequencing libraries were prepared by using the DNAPrep Kit (Illumina, https://www.illumina.com) and a custom xGEN NGS Hybridization Capture DNA panel (Integrated DNA Technologies, https://www.idtdna.com) based on MXPV 2022/MA001 strain sequence (GenBank accession no. ON563414.2). Libraries were sequenced on Nextseq 2000 or NovaSeq 6000 (Illumina) instruments by using 2 × 150-bp kits targeting ≥1 million reads per sample.

Bioinformatic Analysis

Paired-end raw reads were adaptor- and quality-trimmed with Trimmomatic version 0.39 (www.usadellab.org/cms/?page=trimmomatic). Unpaired reads and reads shorter than 120-bp were discarded. Trimmed reads were aligned to the West Africa MPXV reference strain (GenBank accession no. NC_063383.1) by using bbmap version 38.96 (https://github.com/BioInfoTools/BBMap), and duplicate reads were discarded. Ambiguously mapped reads were randomly assigned to one of the top-scoring sites to give the inverted terminal repeats regions even coverage. The consensus genome was generated by 3 iterations of consensus calling using Samtools mpileup version 1.15 (https://www.htslib.org/doc/samtools-mpileup.html) and iVar consensus version 1.3.1(https://andersen-lab.github.io/ivar/html/manualpage.html) with a minimum base quality of 15, a minimum frequency threshold of 0.6, and a minimum depth of 5, then remapped reads to the most recent

consensus. After each iteration, any leading or trailing ambiguous bases (Ns) were removed by using Revica (https://github.com/greninger-lab/revica).

Phylogenetic Placement on Global MPXV Tree

We used the UShER tool (https://github.com/bpt26/USHER_ANALYSES) to place our sequences on a global tree of all available MPXV genomes at the time of writing via the web interface (https://genome.ucsc.edu/cgi-bin/hgPhyloPlace) with default settings. We visualized the resulting subtrees using Nextstrain/Auspice (Appendix Figure 3).

Appendix Table. Sequencing data obtained during genomic analysis of early monkeypox virus outbreak strains, Washington State, United States*

Isolate Name	GenBank accession no.	SRA accession no.	Patient no.†	Lineage
MpxV/human/USA/WA-UW-083698/2022	OP442945.1	SRR21524973	P01	B.1
MpxV/human/USA/WA-UW-088793/2022	OP442947.1	SRR21524979	P01	B.1
MpxV/human/USA/WA-UW-083781/2022	OP392544.1	SRR21524974	P02	B.1
MpxV/human/USA/WA-UW-085171/2022	OP392546.1	SRR21524961	P02	B.1
MpxV/human/USA/WA-UW-087006/2022	OP257260.1	SRR21236084	P03	B.1
MpxV/human/USA/WA-UW-088092/2022	OP392551.1	SRR21524968	P03	B.1
MpxV/human/USA/WA-UW-086040/2022	OP257258.1	SRR21236086	P04	B.1.3
MpxV/human/USA/WA-UW-082770/2022	OP392540.1	SRR21524969	P04	B.1.3
MpxV/human/USA/WA-UW-083953/2022	OP328307.1	SRR21236131	P05	B.1
MpxV/human/USA/WA-UW-087336/2022	OP392550.1	SRR21524987	P05	B.1
MpxV/human/USA/WA-UW-087301/2022	OP310047.1	SRR21236139	P06	B.1
MpxV/human/USA/WA-UW-084148/2022	OP392545.1	SRR21524986	P06	B.1
MpxV/human/USA/WA-UW-074949/2022	OP184762.1	SRR20973038<br>**SRR21616210**	P07	B.1.3
MpxV/human/USA/WA-UW-074988/2022	OP184765.1	SRR20973035<br>**SRR21616209**	P07	B.1.2
MpxV/human/USA/WA-UW-073669/2022	OP123049.1	SRR20736989	P08	B.1.3
MpxV/human/USA/WA-UW-076854/2022	OP123050.1	SRR20736988	P08	B.1
MpxV/human/USA/WA-UW-076773/2022	OP184763.1	SRR20973037	P09	B.1.1
MpxV/human/USA/WA-UW-074932/2022	OP184764.1	SRR20973036	P09	B.1.1
MpxV/human/USA/WA-UW-071121/2022	OP123047.1	SRR20736991	P10	B.1
MpxV/human/USA/WA-UW-076861/2022	OP123048.1	SRR20736990	P10	B.1
MpxV/human/USA/WA-UW-087424/2022	OP257248.1	SRR21236101	P11	B.1.2
MpxV/human/USA/WA-UW-085462/2022	OP392547.1	SRR21524972	P11	B.1.2
MpxV/human/USA/WA-UW-073909/2022	OP055800.1	SRR20653196	NA	B.1.1
MpxV/human/USA/WA-UW-076724/2022	OP055804.1	SRR20653192	NA	B.1
MpxV/human/USA/WA-UW-079141/2022	OP055806.1	SRR20653190	NA	B.1
MpxV/human/USA/WA-UW-074372/2022	OP055807.1	SRR20653189	NA	B.1
MpxV/human/USA/WA-UW-075986/2022	OP055808.1	SRR20653188	NA	B.1
MpxV/human/USA/WA-UW-076082/2022	OP055809.1	SRR20653187	NA	B.1
MpxV/human/USA/WA-UW-073974/2022	OP123040.1	SRR20731575	NA	B.1
MpxV/human/USA/WA-UW-070134/2022	OP123041.1	SRR20731574	NA	B.1.1
MpxV/human/USA/WA-UW-077622/2022	OP123042.1	SRR20731573	NA	B.1
MpxV/human/USA/WA-UW-077836/2022	OP123043.1	SRR20731572	NA	B.1
MpxV/human/USA/WA-UW-071246/2022	OP123044.1	SRR20736996	NA	B.1
MpxV/human/USA/WA-UW-076225/2022	OP123045.1	SRR20736995	NA	B.1.2
MpxV/human/USA/WA-UW-078870/2022	OP123046.1	SRR20736992	NA	B.1.1
MpxV/human/USA/WA-UW-070499/2022	OP169336.1	SRR20913445	NA	B.1
MpxV/human/USA/WA-UW-074224/2022	OP169340.1	SRR20913438	NA	B.1
MpxV/human/USA/WA-UW-074184/2022	OP169341.1	SRR20913437	NA	B.1.1
MpxV/human/USA/WA-UW-071966/2022	OP169344.1	SRR20913433	NA	B.1.1
MpxV/human/USA/WA-UW-075687/2022	OP169345.1	SRR20913443	NA	B.1.3
MpxV/human/USA/WA-UW-072839/2022	OP169346.1	SRR20913442	NA	B.1.1
MpxV/human/USA/WA-UW-079401/2022	OP184760.1	SRR20973040	NA	B.1
MpxV/human/USA/WA-UW-078603/2022	OP184761.1	SRR20973039	NA	B.1
MpxV/human/USA/WA-UW-089015/2022	OP257243.1	SRR21236108	NA	B.1.2
MpxV/human/USA/WA-UW-080268/2022	OP257244.1	SRR21236106	NA	B.1
MpxV/human/USA/WA-UW-084909/2022	OP257245.1	SRR21236104	NA	B.1
MpxV/human/USA/WA-UW-083578/2022	OP257246.1	SRR21236103	NA	B.1
MpxV/human/USA/WA-UW-088432/2022	OP257247.1	SRR21236102	NA	B.1

Isolate Name	GenBank accession no.	SRA accession no.	Patient no.†	Lineage
MpxV/human/USA/WA-UW-081786/2022	OP257249.1	SRR21236100	NA	B.1
MpxV/human/USA/WA-UW-087527/2022	OP257250.1	SRR21236099	NA	B.1
MpxV/human/USA/WA-UW-081272/2022	OP257251.1	SRR21236096	NA	B.1.1
MpxV/human/USA/WA-UW-085937/2022	OP257252.1	SRR21236095	NA	B.1
MpxV/human/USA/WA-UW-088081/2022	OP257253.1	SRR21236094	NA	B.1
MpxV/human/USA/WA-UW-080706/2022	OP257257.1	SRR21236088	NA	B.1.1
MpxV/human/USA/WA-UW-085684/2022	OP257259.1	SRR21236085	NA	B.1
MpxV/human/USA/WA-UW-084325/2022	OP257261.1	SRR21236083	NA	B.1
MpxV/human/USA/WA-UW-087619/2022	OP257262.1	SRR21236082	NA	B.1.3
MpxV/human/USA/WA-UW-089987/2022	OP257263.1	SRR21236081	NA	B.1
MpxV/human/USA/WA-UW-080976/2022	OP257264.1	SRR21236080	NA	B.1
MpxV/human/USA/WA-UW-089183/2022	OP257265.1	SRR21236078	NA	B.1
MpxV/human/USA/WA-UW-083179/2022	OP257266.1	SRR21236077	NA	B.1
MpxV/human/USA/WA-UW-084449/2022	OP257267.1	SRR21236142	NA	B.1
MpxV/human/USA/WA-UW-080617/2022	OP310039.1	SRR21236109	NA	B.1
MpxV/human/USA/WA-UW-088656/2022	OP310040.1	SRR21236098	NA	B.1
MpxV/human/USA/WA-UW-080185/2022	OP310041.1	SRR21236087	NA	B.1.1
MpxV/human/USA/WA-UW-089266/2022	OP310042.1	SRR21236076	NA	B.1.1
MpxV/human/USA/WA-UW-086841/2022	OP310043.1	SRR21236075	NA	B.1
MpxV/human/USA/WA-UW-083888/2022	OP310044.1	SRR21236074	NA	B.1
MpxV/human/USA/WA-UW-083914/2022	OP310046.1	SRR21236140	NA	B.1.2
MpxV/human/USA/WA-UW-088871/2022	OP310048.1	SRR21236138	NA	B.1
MpxV/human/USA/WA-UW-082029/2022	OP310051.1	SRR21236135	NA	B.1
MpxV/human/USA/WA-UW-081935/2022	OP310052.1	SRR21236134	NA	B.1
MpxV/human/USA/WA-UW-082670/2022	OP310053.1	SRR21236133	NA	B.1
MpxV/human/USA/WA-UW-088213/2022	OP310054.1	SRR21236132	NA	B.1.1
MpxV/human/USA/WA-UW-086360/2022	OP310055.1	SRR21236130	NA	B.1.1
MpxV/human/USA/WA-UW-089437/2022	OP310056.1	SRR21236129	NA	B.1
MpxV/human/USA/WA-UW-089714/2022	OP310057.1	SRR21236128	NA	B.1
MpxV/human/USA/WA-UW-087349/2022	OP310058.1	SRR21236127	NA	B.1.1
MpxV/human/USA/WA-UW-088764/2022	OP310061.1	SRR21236124	NA	B.1.1
MpxV/human/USA/WA-UW-089836/2022	OP310062.1	SRR21236123	NA	B.1
MpxV/human/USA/WA-UW-082810/2022	OP310063.1	SRR21236122	NA	B.1.1
MpxV/human/USA/WA-UW-082497/2022	OP310064.1	SRR21236121	NA	B.1
MpxV/human/USA/WA-UW-088973/2022	OP310065.1	SRR21236119	NA	B.1.3
MpxV/human/USA/WA-UW-089152/2022	OP310066.1	SRR21236118	NA	B.1
MpxV/human/USA/WA-UW-082625/2022	OP310067.1	SRR21236116	NA	B.1
MpxV/human/USA/WA-UW-088225/2022	OP310068.1	SRR21236105	NA	B.1
MpxV/human/USA/WA-UW-088152/2022	OP310069.1	SRR21236079	NA	B.1
MpxV/human/USA/WA-UW-083083/2022	OP328308.1	SRR21236115	NA	B.1
MpxV/human/USA/WA-UW-089142/2022	OP328309.1	SRR21236114	NA	B.1
MpxV/human/USA/WA-UW-088725/2022	OP328310.1	SRR21236112	NA	B.1
MpxV/human/USA/WA-UW-088258/2022	OP328311.1	SRR21236111	NA	B.1
MpxV/human/USA/WA-UW-087564/2022	OP328312.1	SRR21236110	NA	B.1
MpxV/human/USA/WA-UW-080100/2022	OP392535.1	SRR21524985	NA	B.1
MpxV/human/USA/WA-UW-080292/2022	OP392536.1	SRR21524966	NA	B.1
MpxV/human/USA/WA-UW-080834/2022	OP392537.1	SRR21524964	NA	B.1.3
MpxV/human/USA/WA-UW-081469/2022	OP442944.1	SRR21524980	NA	B.1.3
MpxV/human/USA/WA-UW-081714/2022	OP392538.1	SRR21524978	NA	B.1
MpxV/human/USA/WA-UW-082215/2022	OP392539.1	SRR21524967	NA	B.1.3
MpxV/human/USA/WA-UW-082488/2022	OP442941.1	SRR21236117	NA	B.1.8
MpxV/human/USA/WA-UW-082880/2022	OP392541.1	SRR21524977	NA	B.1
MpxV/human/USA/WA-UW-083506/2022	OP392542.1	SRR21524981	NA	B.1
MpxV/human/USA/WA-UW-083584/2022	OP392543.1	SRR21524963	NA	B.1
MpxV/human/USA/WA-UW-084331/2022	OP442942.1	SRR21236107	NA	B.1.4
MpxV/human/USA/WA-UW-085393/2022	OP442946.1	SRR21524975	NA	B.1
MpxV/human/USA/WA-UW-086026/2022	OP442943.1	SRR21236097	NA	B.1.8
MpxV/human/USA/WA-UW-087094/2022	OP392548.1	SRR21524960	NA	B.1
MpxV/human/USA/WA-UW-087104/2022	OP392549.1	SRR21524976	NA	B.1.1
MpxV/human/USA/WA-UW-088325/2022	OP392552.1	SRR21524971	NA	B.1
MpxV/human/USA/WA-UW-088960/2022	OP392553.1	SRR21524982	NA	B.1.4

*Sequence data are available in GenBank in the National Center for Biotechnology Information (https://www.ncbi.nlm.nih.gov); raw reads have been deposited to the NCBI sequence read archive (SRA) under BioProject no. PRJNA862948. Bold text indicates resequencing was performed to confirm coinfection and these reads were deposited in separate SRRs.

†Patient numbers are indicated for sequences obtained from the same individual and correspond to tip labels on the phylogenetic tree in Figure, panel A.

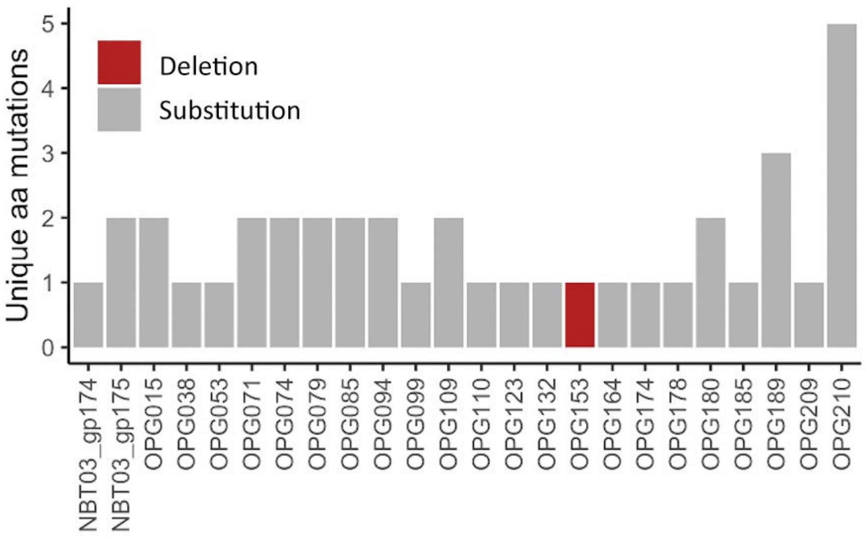

Appendix Figure 1. Unique amino acid (aa) mutations found during phylogenetic analysis of 109 genomes from early monkeypox virus outbreak, Washington, United States. After excluding genes with mutations (amino acid substitutions or deletions) in a single sample, most genes contained 1–2 mutations, except OPG189 and OPG210.

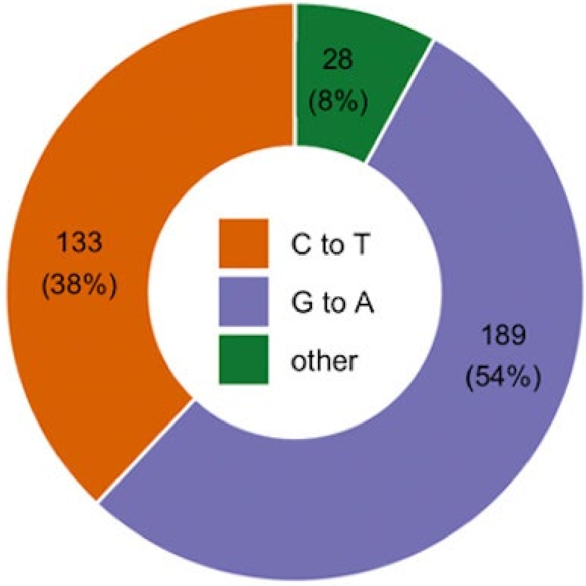

Appendix Figure 2. Distribution of mutations found during phylogenetic analysis of 109 genomes from early monkeypox virus outbreak, Washington, United States. We noted an abundance of G to A and C to T mutations, indicating likely apolipoprotein B mRNA editing catalytic polypeptide-like3 involvement, as reported by C.M. Gigante, et al., https://doi.org/10.1126/science.add4153.

A

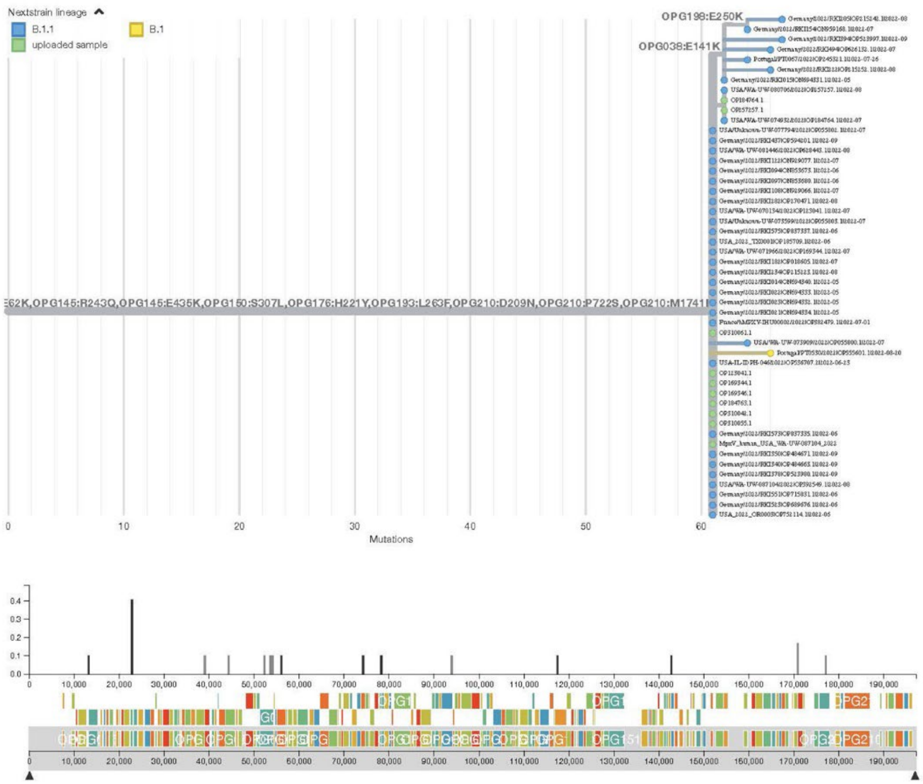
Nextstrain lineage
B.1.1
B.1
uploaded sample
OPG198:E250K
OPG038:E141K
E62K,OPG145:R243Q,OPG145:E435K,OPG150:S307L,OPG176:H221Y,OPG193:L263F,OPG210:D209N,OPG210:P722S,OPG210:M1741I
Mutations

B

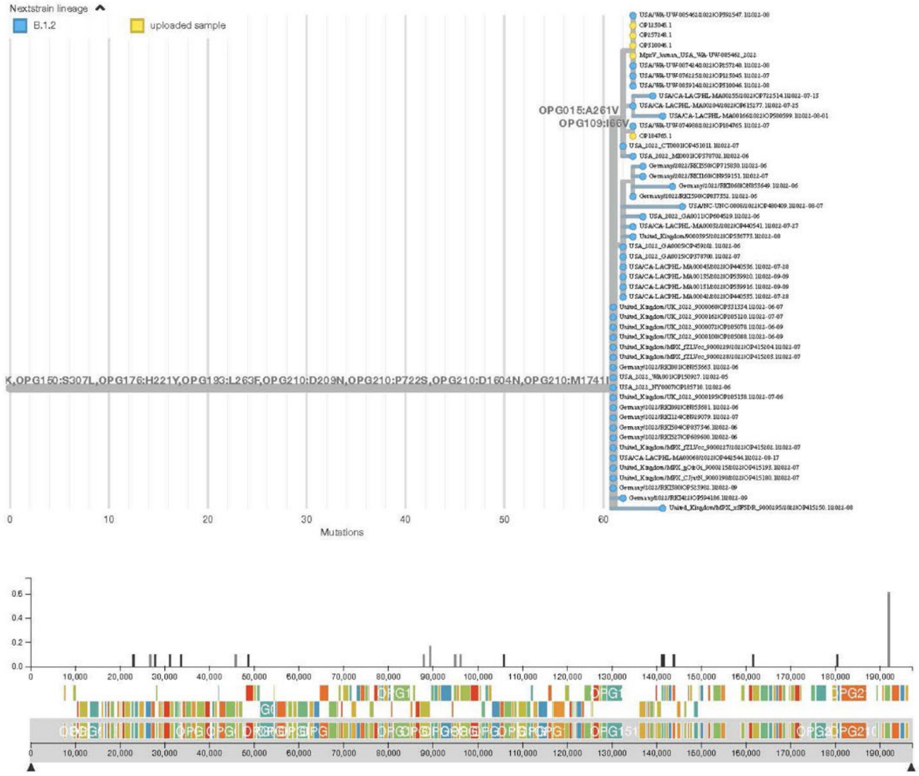
Nextstrain lineage
B.1.2
uploaded sample
OPG015:A261V
OPG109:I66V
K,OPG150:S307L,OPG176:H221Y,OPG193:L263F,OPG210:D209N,OPG210:P722S,OPG210:D1604N,OPG210:M1741I
Mutations

C

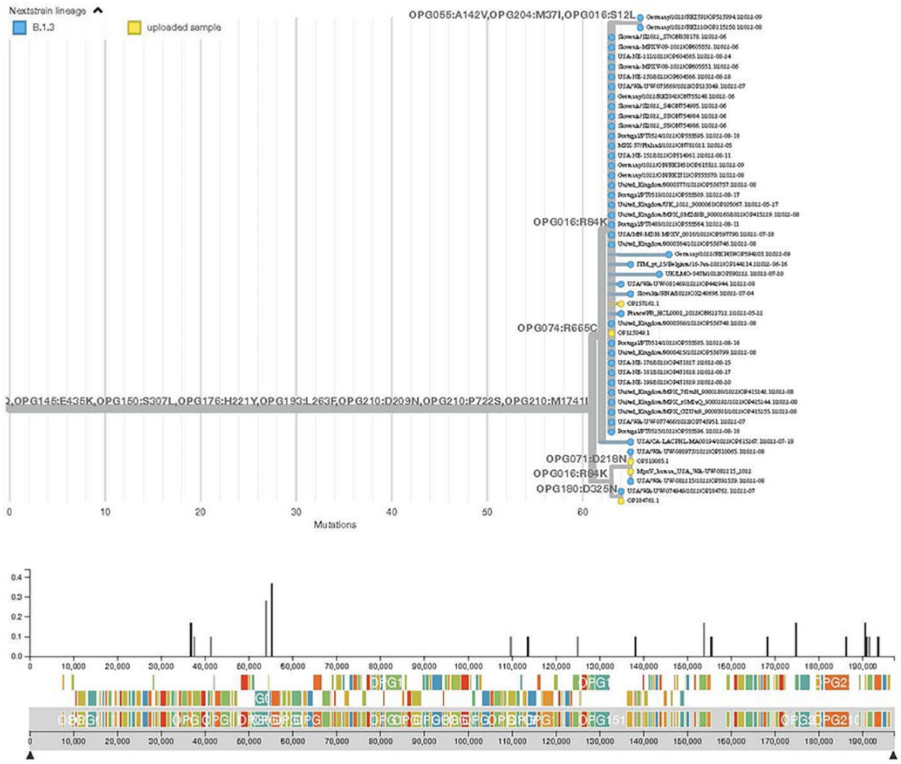
Nextstrain lineage
B.1.3
uploaded sample
OPG055:A142V,OPG204:M37I,OPG016:S12L
OPG016:R84K
OPG074:R665C
OPG145:E435K,OPG150:S307L,OPG176:H221Y,OPG193:L263F,OPG210:D209N,OPG210:P722S,OPG210:M1741I
OPG071:D218N
OPG016:R84K
OPG180:D325N
0
10
20
30
40
50
60
Mutations

D

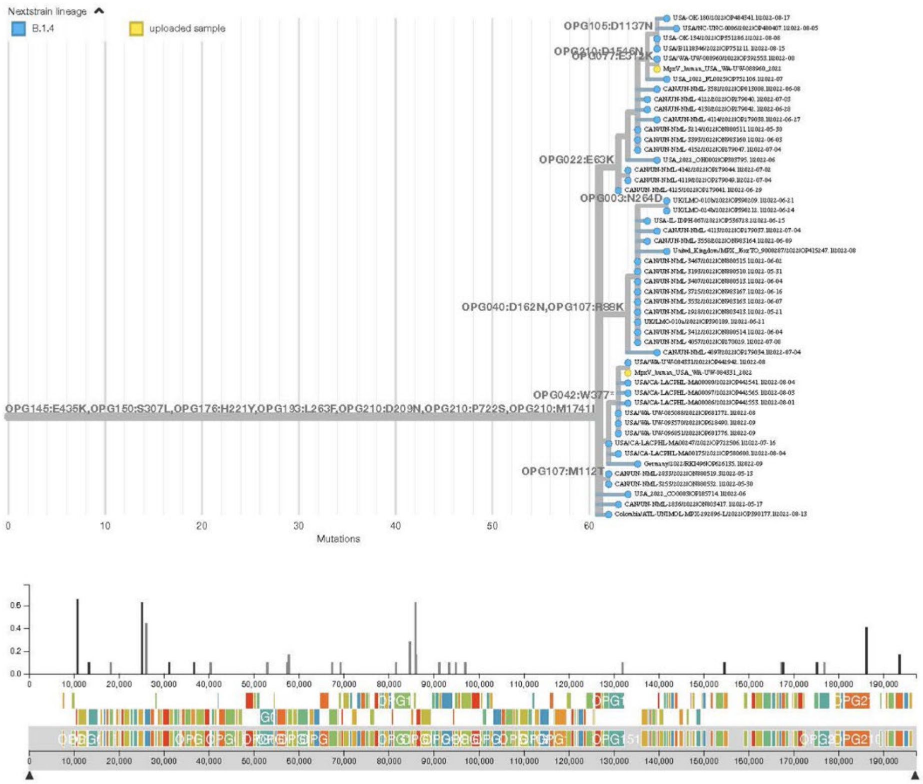
Nextstrain lineage
B.1.4
uploaded sample
OPG105:D1137N
OPG022:E63K
OPG003:N264D
OPG040:D162N,OPG107:R88K
OPG042:W377*
OPG145:E435K,OPG150:S307L,OPG176:H221Y,OPG193:L263F,OPG210:D209N,OPG210:P722S,OPG210:M1741I
OPG107:M112T
0
10
20
30
40
50
60
Mutations

E

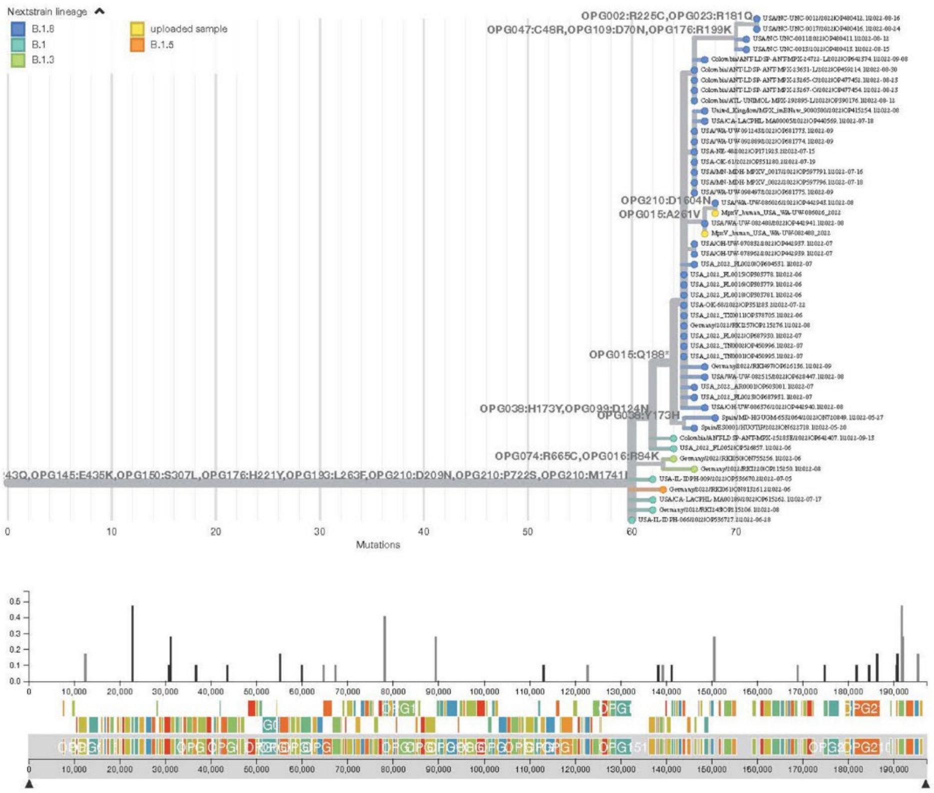

Appendix Figure 3. UShER subtrees for sequences from phylogenetic analysis of early monkeypox virus outbreak, Washington, United States. The trees show mpox lineages from 109 genomes from Washington and nearest neighbor sequences from a global phylogeny. A) B.1.1; B) B.1.2; C) B.1.3; D) B.1.4; E) B.1.8. Subtrees were visualized using Auspice (https://www.auspice.us).
